# The Non-Canonical Iron-Responsive Element of IRE-tvcp12 Hairpin Structure at the 3′-UTR of *Trichomonas vaginalis* TvCP12 mRNA That Binds TvHSP70 and TvACTN-3 Can Regulate mRNA Stability and Amount of Protein

**DOI:** 10.3390/pathogens12040586

**Published:** 2023-04-12

**Authors:** Claudia R. León-Sicairos, Elisa E. Figueroa-Angulo, Jaeson S. Calla-Choque, Rossana Arroyo

**Affiliations:** Department of Infectomics and Molecular Pathogenesis, Center of Research and Advanced Studies of IPN (CINVESTAV-IPN), Av. IPN # 2508, Col. San Pedro Zacatenco, Mexico City 07360, Mexico; claudialeonsicairos@uas.edu.mx (C.R.L.-S.); jcalla@health.ucsd.edu (J.S.C.-C.)

**Keywords:** atypical RNA-binding proteins, TvCP12, non-canonical iron-responsive element-tvcp12 (IRE-tvcp12), IRE/IRP system, mRNA stability, Northwestern blot assay, posttranscriptional iron regulation, REMSA, *Trichomonas vaginalis*, UV cross-linking assay

## Abstract

*Trichomonas vaginalis* is one of the most common sexually transmitted parasites in humans. This protozoan has high iron requirements for growth, metabolism, and virulence. However, iron concentrations also differentially modulate *T. vaginalis* gene expression as in the genes encoding cysteine proteinases TvCP4 and TvCP12. Our goal was to identify the regulatory mechanism mediating the upregulation of *tvcp12* under iron-restricted (IR) conditions. Here, we showed by RT-PCR, Western blot, and immunocytochemistry assays that IR conditions increase mRNA stability and amount of TvCP12. RNA electrophoretic mobility shift assay (REMSA), UV cross-linking, and competition assays demonstrated that a non-canonical iron-responsive element (IRE)-like structure at the 3′-untranslated region of the *tvcp12* transcript (IRE-tvcp12) specifically binds to human iron regulatory proteins (IRPs) and to atypical RNA-binding cytoplasmic proteins from IR trichomonads, such as HSP70 and α-Actinin 3. These data were confirmed by REMSA supershift and Northwestern blot assays. Thus, our findings show that a positive gene expression regulation under IR conditions occurs at the posttranscriptional level possibly through RNA-protein interactions between atypical RNA-binding proteins and non-canonical IRE-like structures at the 3′-UTR of the transcript by a parallel mechanism to the mammalian IRE/IRP system that can be applied to other iron-regulated genes of *T. vaginalis.*

## 1. Introduction

Iron is a cofactor in many biochemical activities. In several enzymes, iron is necessary to carry on cellular processes, including energy-producing redox reactions, oxygen transport, DNA synthesis, and cellular detoxification [1]. However, in excess, iron leads to the generation of free radicals through the Fenton reaction that causes protein, DNA, and lipid damage. Thus, the cell iron homeostasis regulates the iron uptake, storage, and elimination mainly at the posttranscriptional level [2].

The posttranscriptional iron regulation is mediated by the IRE/IRP system, by RNA-protein interactions between *trans*-acting cytoplasmic iron regulatory proteins (IRP-1 and IRP-2) with *cis*-acting iron-responsive elements (IREs). IREs are stem-loop structures present within the 5′- or 3′-untranslated regions (UTRs) of some mRNAs [3,4,5,6]. The regulatory outcome depends on the IRE position in the mRNA. In iron-restricted (IR) conditions, if the IRE is in the 5′-UTR, this IRP protein interaction causes a translational blockage. However, if the IRE is in the 3′-UTR, this interaction promotes mRNA stability by suppressing mRNA degradation, and the protein is synthesized [2]. In the high iron (HI) concentration, the IRP-1 binds to a 4Fe–4S cluster and its RNA-binding ability is lost, functioning as a cytosolic aconitase enzyme. In contrast, the IRP-2 is targeted for proteasomal degradation [7,8,9].

*Trichomonas vaginalis* causes trichomoniasis, the number one non-viral sexually transmitted neglected infection worldwide [10]. This protozoan has multiple cysteine proteinases (CPs) mainly of the papain superfamily [11,12]. CPs are important virulence factors in *T. vaginalis* participating in cytoadherence, cytotoxicity, hemolysis, induction of apoptosis, and IgG digestion [13,14,15,16,17,18,19,20,21,22,23], among others [24,25]. Expression analysis of some CPs, such as TvCP2, TvCP4, TvCP12, TvCP39, and TvCP65 reveals that these CPs are differentially modulated by iron [12,17,19,23,26,27,28,29].

Iron is an essential nutrient for growth, metabolism, virulence, and antigen expression in parasitic protozoa, including *T. vaginalis* [17,26,27,30,31,32]. However, knowledge of the molecular mechanisms controlling gene expression by iron at the transcriptional and posttranscriptional levels in trichomonads is still limited. At the transcriptional level, an iron-responsive promoter and multiple DNA elements bind different proteins that are found to be responsible for the iron-inducible transcription of the *ap65-1* adhesin gene [33,34,35,36,37,38]. An iron regulatory mechanism controlling gene expression at the posttranscriptional level parallel to the IRE/IRP system has also been reported in this parasite. The positive iron regulation of the trichomonad TvCP4 is due to the presence of a non-canonical IRE-like hairpin stem-loop structure at the 5′ region of the *tvcp4* mRNA (IRE-tvcp4) [29]. Its interaction with atypical cytoplasmic RNA-binding proteins of *T. vaginalis* in iron-restricted (IR) conditions, blocking its translation by an IRE/IRP-like system, has been described [29,39,40]. However, *T. vaginalis* lacks aconitase activity or IRPs. Instead, the IRE-tvcp4 hairpin could specifically interact with at least four cytoplasmic proteins of 135, 110, 65, and 45 kDa. Two of these proteins have been previously characterized as atypical RNA-binding proteins, such as the α-actinin 3 (TvACTN3) and the chaperone HSP70 (TvHSP70) [39,40].

We previously demonstrated that the *tvcp12* transcript is downregulated by iron possibly at the posttranscriptional level due to differences in the length of the poly-A tail mRNA [28]. Our goal in this work was to identify the mechanism of iron regulation at the posttranscriptional level. Our new findings demonstrate that the binding of atypical RNA-binding proteins (TvACTN3 and TvHSP70) to a non-canonical IRE-like structure found at the 3′-UTR of the *tvcp12* transcript (IRE-tvcp12) can positively regulate its expression by iron-restriction by increasing mRNA stability and the amount of TvCP12. These data show that the 3′-UTR IRE-tvcp12 plays a key role in the differential iron posttranscriptional regulation of the *tvcp12* gene.

## 2. Materials and Methods

### 2.1. Parasites and Culture Conditions

*T. vaginalis* parasites of the isolate CNCD 147 were grown in trypticase-yeast extract-maltose (TYM) medium supplemented with 10% (*v*/*v*) heat-inactivated horse serum [41] (~20 μΜ iron; normal: N) [26]. Parasites were also grown under high (HI) or IR medium by adding into the TYM medium of 250 μΜ ammonium ferrous sulfate, or 150 μΜ of 2-2′dipyridyl (Sigma-Aldrich, Co., St. Louis, MO, USA), respectively, as previously reported [26,31].

### 2.2. RT-PCR Assays

Semi-quantitative RT-PCR assays were performed using the same primers and conditions reported previously [28] with modifications. In this case, both *tvcp12* and *β-tubulin* primers were used in the same reaction. Briefly, the total RNA obtained using TRIzol reagent (Sigma) from trichomonads grown under IR and HI conditions was reversed-transcribed using an oligo (dT) primer. Amplicons of 270 and 112 bp of the trichomonad *tvcp12* and *β-tubulin* cDNAs, respectively, were obtained by PCR using the same primers previously reported [28,42]. The *β-tubulin* was used as an internal control [28,42].

### 2.3. Tvcp12 mRNA Stability Assays

Total RNA from HI and IR trichomonads were obtained at 0, 1, 3, 6, 12, and 24 h after actinomycin D-induced transcriptional blockage using parasites in the semi-logarithmic phase (12 h growth after inoculation) and 50 µg/mL actinomycin D (Sigma-Aldrich). *Tvcp12* and *β-tubulin* mRNAs were measured by multiplex RT-PCR, as described below. The bands’ intensity in ethidium bromide-stained agarose gels was quantified by densitometric analysis using the Quantity One program (Bio-Rad, Hercules, CA, USA). The pixels of tubulin amplicons in HI and IR trichomonads without treatment (t_0_) were taken as 100% in each condition to normalize *tvcp12* mRNA levels. Experimental *tvcp12* mRNA half-life was determined by plotting the *tvcp12* mRNA amount at different times on a semi-logarithmic scale, taking the *tvcp12* mRNA amount at t_0_ as 100% in each condition. The theoretical half-life of the *tvcp12* mRNA was obtained by using the decay equation t_1/2_ = ln 2/K, where K corresponds to the decay constant [43].

### 2.4. Western Blotting Assays

Total trichomonad proteins from 2 × 10^7^ parasites grown in HI, N, and IR medium obtained by 10% trichloroacetic acid (TCA) precipitation as before [26], were separated by SDS-PAGE using 12% polyacrylamide gels and Coomassie brilliant blue (CBB) stained. For Western blot analysis, duplicated gels were transferred onto a nitrocellulose (NC) membrane to immunodetect TvCP12 with a mouse polyclonal antibody (α-pepTvCP12, 1:1000 dilution) generated against a synthetic peptide (DVSKGYAKVT) selected from the most divergent region (residues 201–210) of the TvCP12 proteinase (TVAG_410260) by multiple alignments, comparing it with the other papain-like cysteine proteinases reported in the *T. vaginalis* genome database TrichDB [11]. A pre-immune normal mouse serum (NMS, 1:1000 dilution) was used as a negative control. In addition to the CBB-stained pattern of total protein extracts shown, an anti-α-tubulin monoclonal antibody (1:100 dilution) against chicken brain tubulin (Zymed, San Francisco, CA, USA) was also used as a quantity control. Western blots were developed by chemiluminescence using the ECL-Plus kit (Amersham).

### 2.5. Immunocytochemical Assay

To localize TvCP12 in trichomonads, immunocytochemical assays were performed by the Avidin-Biotin Complex Histostatin-SP kit (ABC, Zymed) system as recommended by the manufacturer. Fixed and permeabilized or non-permeabilized HI, N, and IR parasites prepared as before [26] were incubated at the same dilution (1:50) with the α-pepTvCP12 antibody or with the NMS used as a negative control.

### 2.6. Purification of Recombinant Proteins

The human IRP-1 protein (hIRP-1r) subcloned into the pGEX plasmid, containing the full-length cDNA (kindly donated by Dr. Lukas Kühn) and the trichomonad actinin-3 (TvACTN-3r), heat-shock protein 70 (TvHSP70r), and triose phosphate isomerase-2 (TvTIM-2r) were expressed and purified by affinity chromatography as before [29,39,40]. Purified proteins were quantified by Bradford (Bio-Rad) and their purity was assessed by SDS-PAGE on 10% polyacrylamide CBB-stained gels. The hIRP-1r and TvTIM2r proteins were used as positive or negative controls, respectively. TvACTN-3r and TvHSP70r were used as reagents for RNA electrophoretic mobility shift assay (REMSA) and UV cross-linking and Northwestern blot (NWB) assays.

### 2.7. Cytoplasmic Extracts from HeLa Cells and Trichomonad Parasites

HeLa cell cytoplasmic extracts were prepared by a modified method [44] and used for REMSA and UV cross-linking assays as previously reported [29,39]. Briefly, HeLa cell pellet was suspended in lysis buffer A, homogenized in a Dounce homogenizer, and centrifuged at 10,000 g for 30 min at 4 °C. Supernatants at 10 mg/mL final concentration were kept at −70 °C for less than two months.

Trichomonad cytoplasmic extracts were prepared from 1 × 10^8^ trichomonads grown in the HI or IR medium by a modified method as previously reported [29,39]. Briefly, parasites were lysed in an interaction buffer, incubated for 20 min at 4 °C, and centrifuged at 13,000 g for 5 min at 4 °C. The supernatant was recovered, protein concentration was determined by Bradford (Bio-Rad), and aliquots were kept at −70 °C for less than three weeks.

### 2.8. In Vitro Transcription of IRE-like Sequences

For in vitro transcription, we used DNA from the pSPT-fer plasmid (a generous gift of Dr. Lukas Kühn) that contains the human ferritin H-chain IRE (IRE-fer) region [45,46]. We also used the following *tvcp12* amplicons: wild-type (wt) IRE-tvcp12 of 19 bp corresponding to the 3′-UTR region after the stop codon of *tvcp12,* an IRE-tvcp12 deletion mutant without the loop sequence, named Dloop, and another IRE-tvcp12 disrupt mutant without half of the stem-loop sequence, named Disrupt, which were produced by PCR using primers: sense 5′-TAATACGACTCACTATAGGGACGTATTTAATTGATTGCG-3′ and antisense 5′-CGCAATCAATTAAATACGT-3′, sense Dloop5′tvcp12 5′-TAATACGACTCACTATAGGGTAAACGTATTATTGCGAAA-3′ and antisense Dloop3′tvcp12 5′-TTTCGCAATAATACGTTA-3′, and sense Disrupt-tvcp12 5′-TAATACGACTCACTATAGGGTAAACGTATTTAATTGAT-3′ and antisense Disrupt-tvcp12 5′-ATCAATTAAATACGTTTA-3′ primers, respectively. The sense primers for PCR contain a bacteriophage T7 promoter sequence (underline nt) and an extra sequence GGG to enhance transcription. For RNA synthesis, we used as templates the purified PCR products and an in vitro transcription kit (Promega, San Luis Obispo, CA, USA). We used DNase RQ1 (Promega) treatment in the presence of RNase inhibitors (Promega) and gel filtration to remove DNA templates and unincorporated nucleotides. For the synthesis of the labeled RNA, transcripts were synthesized by including 20 μCi [α^32^P] UTP (800 Ci/mmol; Dupont) in the transcription reaction. The canonical IRE-fer transcript was used as the positive control [29,39]. The binding of the wt IRE-tvcp12 and the IRE-tvcp12 mutant transcripts to human IRPs and trichomonad atypical RNA-binding proteins was tested by REMSA, UV cross-linking, REMSA supershift, and NWB assays.

### 2.9. RNA Electrophoretic Mobility Shift Assay (REMSA)

RNA-protein complexes (RPCs) were first detected by REMSA as previously reported using ^32^P-labeled RNAs (0.2 ng in 2 × 10^5^ cpm) in 20 μL of reaction volume incubated for 10 min at 25 °C with the recombinant hIRP-1 (1 μg), with HeLa cells (20 μg), or with *T. vaginalis* (50 μg) cytoplasmic extracts in the presence of 5 μg heparin. The RPCs were resolved on a 6% non-denaturing polyacrylamide gel and visualized by autoradiography as before [29,39]. The specificity of the RPC formation was assessed by competition REMSAs performed in the presence of unlabeled RNA (50 and 100-fold molar excesses) and incubated for 30 min at 4 °C. REMSA supershift assays were also performed with 5–15 μL rabbit anti-TvACTN3r, or rabbit anti-TvHSP70r serum, or 10 μL control anti-TvTIM2r serum [47], as a non-related antibody, added to the IR trichomonad cytoplasmic extracts before adding the RNA probes. These experiments were performed at least three independent times, with similar results.

### 2.10. UV Cross-Linking Assays

Briefly, the UV-cross-linking assays were performed as previously reported, by incubating hIRP-1r (0.5–1.0 μg), HeLa cells (30 μg), or *T. vaginalis* (50 μg) cytoplasmic extracts with ^32^P-labeled RNA probes (5 × 10^5^ cpm) for 20 min at 30 °C in 25 μL of a reaction mixture. After, it was irradiated using a UV lamp (240 nm) for 30 min on ice, and then incubated with RNase A (10 μg) and RNase T1 (20 u) for 30 min at 25 °C. Proteins bound to RNA were resolved by SDS-PAGE on 10% polyacrylamide gels, CBB-stained, and dried. The radioactive bands were visualized by autoradiography. For competition assays, a distinct molar excess of the unlabeled IRE-tvcp12, IRE-tvcp4 [29], or IRE-fer transcripts were incubated with cytoplasmic extracts for 15 min at 4 °C before the addition of labeled RNA. tRNA was used as a non-related (NR) RNA [29,39].

### 2.11. Northwestern Blotting (NWB) Assay

The modified Northwestern blot assay was performed as described previously [39] by combining previously described methods [48,49] with a few modifications. In brief, recombinant proteins resolved by 10% SDS-PAGE, were transferred onto NC membranes, blocked with 10% fat-free milk in extraction buffer (EB) for 18 h at 4 °C, washed 3× with EBY at 25 °C, incubated for 18 h at 4 °C with EBY containing 10–15 ng/mL radiolabeled transcripts, and washed 2× with EB and with B buffer. After extensive washing with EB, the NC membranes were washed extensively with EB, dried, and exposed for autoradiography. These experiments were performed independently at least three times, with similar results.

## 3. Results

### 3.1. The tvcp12 mRNA Stability Is Higher in Iron-Restricted Trichomonads

**Figure 1:**
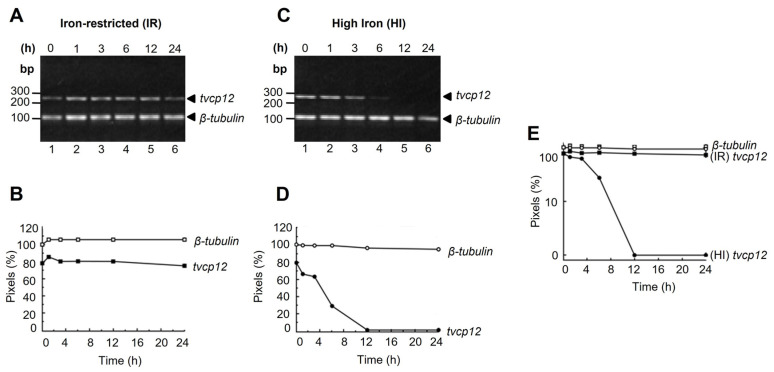
*tvcp12* mRNA stability and half-life under different iron conditions. (**A**,**C**) Lanes 1 to 6, ethidium bromide-stained gels showing the duplex RT-PCR products obtained at different times (0, 1, 3, 6, 12, and 24 h) after the actinomycin D treatment. (**B**,**D**) Densitometric analysis of RT-PCR products shown in (**A**,**C**). (**E**) The semi-logarithmic plot of the *tvcp12* and *β-tubulin* mRNA levels quantified by densitometry in (**A**,**C**). (○) control of *β-tubulin* in high iron (HI) conditions; (●) *tvcp12* in HI conditions; (□) control of *β-tubulin* in iron-restricted (IR) conditions; (■) *tvcp12* in IR conditions. Experiments were performed three times with similar results.

By Northern blot and RT-PCR assays, we previously demonstrated that the *tvcp12* transcript increased in IR compared to in HI conditions [28]. To identify the iron regulatory mechanism controlling the expression of *tvcp12*, we first investigated whether iron regulates the amount of *tvcp12* transcript by modulating mRNA stability. The *tvcp12* mRNA was chased after a transcriptional blockage in actinomycin D-treated trichomonads grown under different iron conditions and measured at 0 to 24 h by semi-quantitative duplex RT-PCR assays using total RNA. We also amplified the *β-tubulin* mRNA as an internal control. Figure 1 shows that the amount of the *tvcp12* mRNA in IR trichomonads remained stable even 24 h after the actinomycin D-treatment (Figure 1A,B), while the *tvcp12* mRNA was present in HI-grown trichomonads up to 3 h after the transcriptional blockage (Figure 1C,D). The control *β-tubulin* mRNA showed no major variations after the transcriptional blockage in both iron conditions (Figure 1A,C). In addition, the semi-logarithmic plot of normalized *tvcp12* mRNA levels versus actinomycin D exposure time (Figure 1E) shows that the half-life of the *tvcp12* mRNA from HI trichomonads was ~4 h, while the *tvcp12* mRNA half-life in IR trichomonads was >24 h. These data show that iron concentrations differentially modulate *tvcp12* mRNA stability, which may also affect the amount and localization of TvCP12 in *T. vaginalis*.

### 3.2. The tvcp12 Gene Expression Is Also Upregulated by IR Conditions at the Protein Level

**Figure 2:**
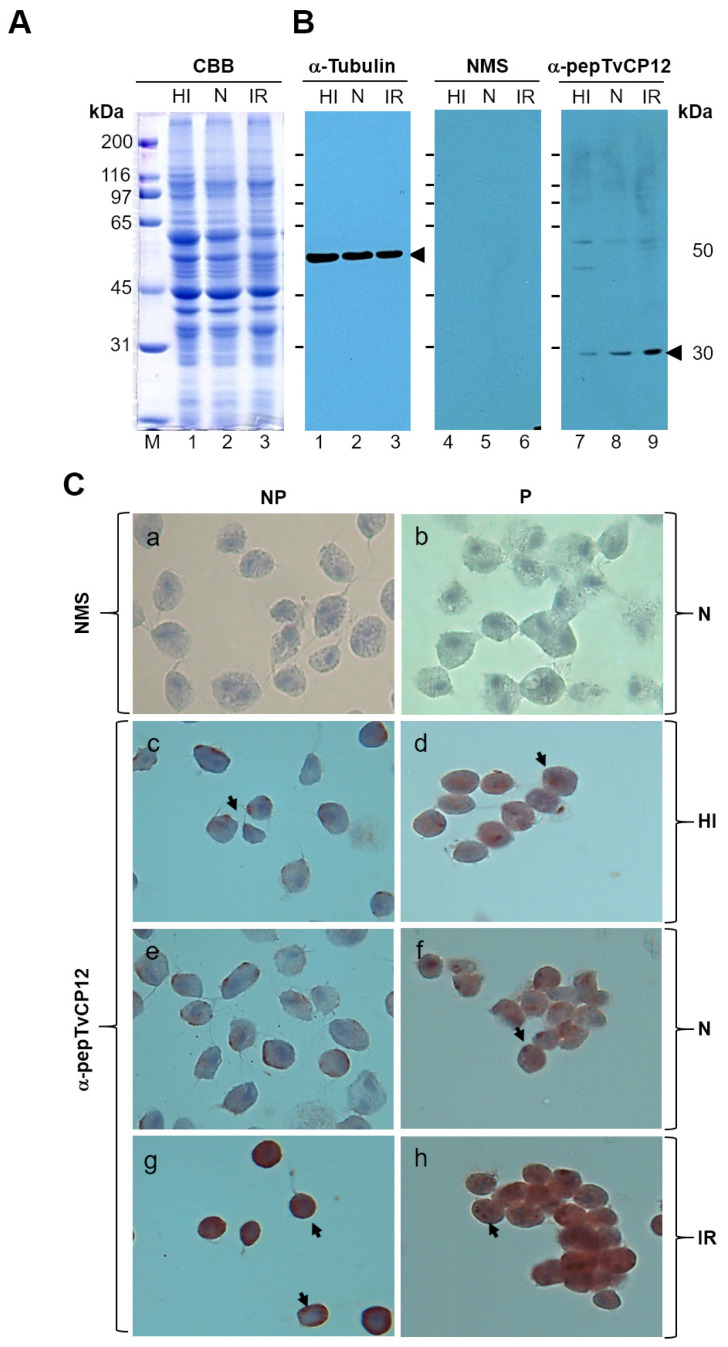
Effect of iron in the expression of *T. vaginalis* TvCP12 proteinase. (**A**,**B**) Western blot with total protein extracts of high iron (HI), normal (N), and iron-restricted (IR) and *T. vaginalis* separated by SDS-PAGE using 10% polyacrylamide gels, which were CBB-stained (**A**) or transferred onto NC membranes (**B**) and incubated with an α-tubulin (lanes 1–3) antibody used as a loading control, preimmune normal mouse serum (NMS) used as a negative control (lanes 4–6), or an α-pepTvCP12 antibody (lanes 7–9). kDa, molecular weight markers in kilodaltons; arrowheads, indicate the size of protein bands detected with the antibodies. (**C**) Effect of iron in the amount and location of *T. vaginalis* TvCP12 proteinase. Chromogenic immunocytochemical assays with the α-pepTvCP12 antibody in fixed non-permeabilized (NP) (a, c, e, and g) or permeabilized (P) (b, d, f, and h) parasites grown in normal (N; a, b, e, and f), high iron (HI; c and d), or iron-restricted (IR; g and h) medium. The NMS was used as a negative control (a and b). Stained parasites were visualized at 100× magnification (Optic microscope, Nikon Instruments Inc., Melville, NY, USA). Arrows point to the parasite surface or cytoplasmic localization of TvCP12. Experiments were performed three independent times with similar results.

We next tested whether the positive IR regulation of the *tvcp12* transcript half-life is also detected at the protein level. Western blot assays with trichomonad total extracts from under different iron conditions (Figure 2A) transferred onto NC membranes using an anti-TvCP12 peptide (α-pepTvCP12) serum antibody were performed. The α-pepTvCP12 antibody showed an increased amount of a 30 kDa band corresponding to TvCP12 in IR extracts (Figure 2B, lane 9, as compared to those from HI and N parasites (Figure 2B, lanes 7 and 8, respectively). While similar amounts of a 50 kDa tubulin protein band used as a loading control were detected in all iron conditions with the anti-tubulin antibody (Figure 2B, lanes 1–3). Immunocytochemistry assays using fixed non-permeabilized (NP) or permeabilized (P) parasites grown in the different iron conditions and an α-pepTvCP12 antibody showed more TvCP12 brown signal on the parasite surface (Figure 2C, panel g) and cytoplasm (Figure 2C, panel h) of IR trichomonads, as compared to HI (Figure 2C panels c and d) and N parasites (Figure 2C, panels e and f), whereas the negative control panels did not show any brown signal, as expected (Figure 2C, panels a and b). These results show that the lack of iron not only increased the amount of transcript due to high mRNA stability (Figure 1) but also increased the expression and surface localization of the TvCP12 protein on *T. vaginalis* (Figure 2).

### 3.3. An IRE-like Structure Was Found at the 3′-UTR of the Tvcp12 mRNA

Thus, to identify the iron regulatory mechanism that modulates the gene expression of TvCP12 at the posttranscriptional level controlling mRNA stability, we searched for IRE-like hairpin-loop structures, at the 3-UTR of the *tvcp12* mRNA that could specifically interact with RNA-binding proteins and participate in a posttranscriptional iron regulatory mechanism [29,50] similar to the conserved eukaryotic IRE/IRP system [3,4,5,6]. 

**Figure 3:**
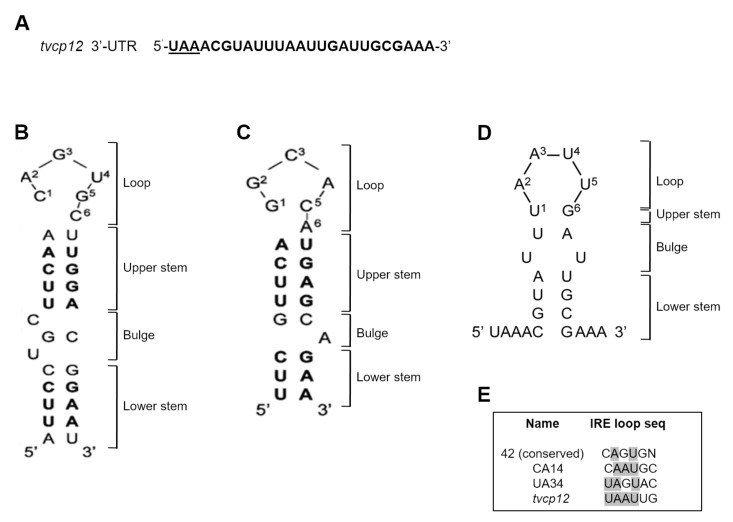
A theoretical model of the 19 nt IRE-tvcp12 hairpin stem-loop structure. (**A**) The 3′-UTR sequence of 25 nt of the *tvcp12* mRNA, including the stop codon (underlined). (**B**) Stem-loop secondary structure of the H-ferritin IRE (IRE-fer) RNA [29]. (**C**) A 31 nt IRE-tvcp4 RNA [29]. (**D**) A theoretical model of the predicted 19 nt IRE-tvcp12 mRNA (5 to 22 nt). (**E**) Comparison among the IRE-**tvcp12** loop and IRE-fer mutant sequences [46]. Gray-shaded nucleotides show the common nucleotides among the known loop sequences. The predicted RNA secondary structure of IRE-**tvcp12** was obtained using the *mfold* program (www.bioinfo.rpi.edu/applications/mfold accessed on 25 October 2004) and confirmed by the RNAfold web server (RNAfold 2.4.18. A) (rna.tbi.univie.ac.at//cgi-bin/RNAWebSuite/RNAfold.cgi?PAGE=3&ID=vMspvxRThL). Reprinted/adapted with permission from Ref. [29]. 2023, John Wiley and Sons’ License Number (5526100025746).

In silico analysis of the *tvcp12* 3′-UTR showed the typical *T. vaginalis* elements for mRNA polyadenylation [51]. Analysis of 25 nt downstream of the UAA stop codon of the *tvcp12* mRNA by the *mfold* program [52] and the RNAfold software [53] predicted the formation of a stem-loop RNA secondary structure (Figure 3D). This sequence formed a stable (Δ*G* −2.5 kcal/mol) 19 nt IRE-like hairpin structure with a 6 nt loop **UAAUUG** (Figure 3D), a 1 nt upper stem, a bubble, and a 4 nt lower stem (Figure 3D). This IRE-like structure may interact with human IRPs and trichomonad atypical cytoplasmic proteins, as the non-canonical IRE-tvcp4 does [29,39,50].

The predicted 19 nt *tvcp12* IRE-like (IRE-tvcp12) hairpin stem-loop RNA secondary structure was compared with the secondary structures of the human IRE-fer [54] (Figure 3B), and with the IRE-tvcp4, an atypical IRE-like structure present in the *tvcp4* mRNA [29,40,50] (Figure 3C). The 6 nt loop of the IRE-tvcp12 structure has a 5′-**UAAUUG**-3′ sequence, which does not correspond to the canonical sequence reported for eukaryotic IRE (Figure 3B) involved in iron homeostasis [3,4,5,6]. It does not correspond to the IRE-tvcp4 sequence either (Figure 3C) [29], but it is similar to mutants CA14 and UA34 of the human ferritin IRE [45] (Figure 3E). However, it has a U1 and G6 nucleotide arrangement, short upper and lower stems, and two unpaired Us, instead of the C bulge that divides the stem regions.

### 3.4. The IRE-tvcp12 Binds to Human IRPs

**Figure 4:**
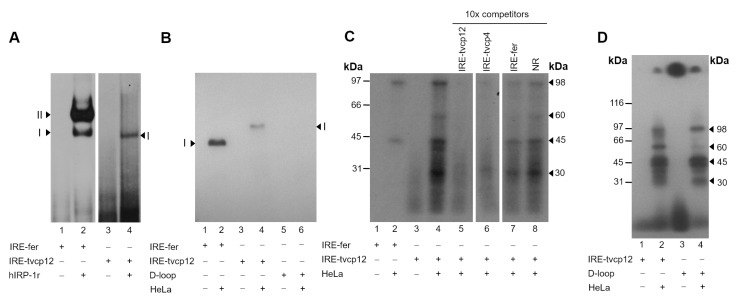
The interaction between the IRE-tvcp12 mRNA structure and human IRPs. (**A**,**B**) Gel-shifting of radiolabeled IRE-fer (control) or IRE-tvcp12, or IRE-tvcp12 D-loop mutant transcripts to hIRP-1r (**A**) and HeLa cytoplasmic extracts (**B**) as described in Experimental procedures. (**A**,**B**) Lanes 1, 3, and 5, free radiolabeled IRE-fer, IRE-tvcp12, or IRE-tvcp12 D-loop mutant transcripts. Lanes 2 and 4, RPCs formed between hIRP-1r or HeLa cytoplasmic extracts and the transcripts IRE-fer and IRE-tvcp12, or (**B**) Lane 6, with the IRE-tvcp12 D-loop mutant transcript. (**A**,**B**) Arrowheads indicate the position of the formed RPCs. (**C**) Specificity of the interaction of the IRE-tvcp12 RNA to HeLa IRP proteins determined by UV cross-linking competition assays performed in the absence (lane 4), or presence of 10-fold molar excess (10×) of unlabeled IRE-tvcp12 (lane 5); IRE-tvcp4 (lane 6), IRE-fer (lane 7), or tRNA (lane 8), as homologous, heterologous, or non-related (NR) RNA competitors, respectively. As a positive control, interaction of IRE-fer to HeLa cytoplasmic extract was used (lane 2). Mock controls without proteins (lanes 1 and 3) were also used as controls. (**D**) UV cross-linking assays to detect the differential interaction of IRE-tvcp12 (lanes 1 and 2) and IRE-tvcp12 D-loop mutant (lanes 3 and 4) with HeLa cytoplasmic extracts. Lanes 1 and 3, radiolabeled free probes. I or II, the position of RPC bands on native gels (**A**,**B**); kDa, molecular weight markers in kilodaltons; arrowheads, indicate the size in kDa of protein bands cross-linked to radiolabeled RNA probes (**C**,**D**) on denaturing gels detected after autoradiography. Experiments were performed three independent times with similar results. (**A**–**D**) (+) or (-) symbols indicate the presence or absence of each component on the REMSA or UV cross-linking reaction.

We tested whether the non-canonical IRE-tvcp12 hairpin found at the 3′-UTR is functional and may modulate the *tvcp12* mRNA stability in response to iron concentrations by interacting with atypical trichomonad cytoplasmic RNA-binding proteins under IR conditions. First, we tested its ability to form RPCs with human IRP proteins by REMSA using a hIRP-1r protein and HeLa cell cytoplasmic extracts (Figure 4). Upon incubation with the radiolabeled IRE-tvcp12 probe, the hIRP-1r showed a single band-shift (Figure 4A, lane 4), as compared with the double shift of the control IRE-fer (Figure 4A, lane 2). As expected, with free RNA probes used as negative controls, no band-shift was observed (Figure 4A, lanes 1 and 3). When the same radiolabeled IRE-tvcp12 probe was incubated with HeLa cytoplasmic extracts, the formation of a single RPC was also observed (Figure 4B, lane 4), as compared with the control IRE-fer probe (Figure 4B, lane 2). As expected, with free RNA transcripts used as negative controls, no band-shift was observed (Figure 4B, lanes 1, 3, and 5). These data show that the IRE-tvcp12 probe containing an IRE-like structure forms at least one RPC with human IRP proteins.

### 3.5. The IRE-tvcp12 Hairpin Specifically Interacts with IRP Proteins from HeLa Cytoplasmic Extracts

Furthermore, to determine the size and specificity of IRP proteins in the RPCs between the IRE-tvcp12 structure and HeLa cytoplasmic IRP proteins, UV cross-linking competition assays with HeLa cytoplasmic extracts and ^32^P-labeled RNA probes: IRE-fer as a positive control and IRE-tvcp12 being tested were performed. Figure 4C shows that 98 and 45 kDa proteins cross-linked with both labeled transcripts (Figure 4C, lanes 2 and 4, respectively). Moreover, 60 and 30 kDa protein bands were also observed with the trichomonad transcript (Figure 4C, lane 4). In mock experiments, as expected, no labeled bands (Figure 4C, lanes 1 and 3) were observed. The 98 kDa protein band corresponds in size to the human IRP-1 present in the cytoplasmic extracts of HeLa cells [29]. As competitors, we used a ten-fold molar excess of unlabeled RNAs: (i) homologous IRE-tvcp12; (ii) heterologous IRE-tvcp4, and (iii) IRE-fer transcripts, and tRNA, as an NR RNA (Figure 4C, lanes 5 to 8). Unlabeled IRE-tvcp12 and IRE-tvcp4 RNAs competed with radiolabeled IRE-tvcp12 transcript (Figure 4C, lanes 5 and 6, respectively) for RPC formation, as compared to the control without competitor (Figure 4C, lane 4), while unlabeled IRE-fer RNA partially cross-competed (up to 50%, Figure 4C, lane 7) and the excess of tRNA had no effect (Figure 4C, lane 8). These data show that these RPCs are specific. Interestingly, these results also show that trichomonad non-canonical IREs are better competitors for RPC formation with HeLa cell extracts under these experimental conditions. 

### 3.6. The Loop Sequence of the IRE tvcp12 Is Necessary for Binding to HeLa IRP Proteins

To further test the relevance of this IRE-tvcp12 structure in the interaction with human IRP proteins, we generated an IRE-tvcp12 deletion mutant without the loop sequence and tested its ability to form RPCs with HeLa cytoplasmic extracts by gel-shifting (Figure 4B) and UV cross-linking assays (Figure 4D). Our results showed that no band shift with the IRE-tvcp12 deletion mutant probe was observed (Figure 4B, lane 6). In addition, the 60 kDa protein was not observed when the deletion mutant was used (Figure 4D, lane 4), as compared with the control IRE-tvcp12 probe (Figure 4D, lane 2). These results suggest that this HeLa protein could be the one recognizing the loop sequence in the trichomonad non-canonical IRE-tvcp12 hairpin.

### 3.7. Effect of Iron on RNA-Protein Complex Formation between the IRE-tvcp12 Hairpin and Trichomonad Cytoplasmic Proteins

**Figure 5:**
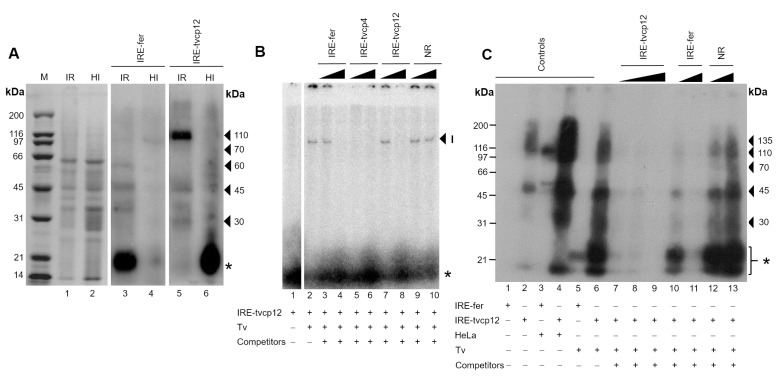
Iron effect on the interaction between the IRE-tvcp12 mRNA and cytoplasmic trichomonad proteins. (**A**) 10% SDS-PAGE polyacrylamide gel CBB-stained cytoplasmic extracts from trichomonads grown under iron-restricted (IR, lane 1) or high iron (HI, lane 2) conditions. UV cross-linking assay was performed to detect the effect of iron on the interaction of IRE-fer (lanes 3 and 4) used as a control IRE; IRE-tvcp12 (lanes 5 and 6) RNA probes with trichomonad cytoplasmic extracts under IR (lanes 3 and 5) or HI conditions (lane 4 and 6). Molecular weight markers (M) in kilodaltons (kDa); arrowheads, indicate the size of protein bands cross-linked to radiolabeled RNA probes on denaturing gels detected after autoradiography; asterisk shows radiolabeled free probe. Experiments were performed three independent times with similar results. (**B**,**C**) Specific interaction between the trichomonad 19 nt IRE-tvcp12 hairpin structure and cytoplasmic extracts of iron-restricted (IR) *T. vaginalis.* Detection of RPCs by (**B**) gel-shifting of ^32^P-IRE-tvcp12 mRNA to parasite cytoplasmic extracts in the absence (lane 2) or presence of 30- and a 100-fold molar excess of unlabeled IRE-fer (lanes 3 and 4), IRE-tvcp4 (lanes 5 and 6), IRE-tvcp12 (lanes 7 and 8), and tRNA (lanes 9 and 10) as heterologous, homologous, or non-related competitors as described in Experimental procedures. Mock control without proteins (lane 1) was also used. Arrowhead shows the band of RPC (I). (**C**) UV cross-linking competition assays performed in the absence (lane 6), or presence of 10-, 20-, and a 100-fold molar excess of unlabeled IRE-tvcp12 transcript (lanes 7 to 9), as a homologous competitor, or 20- and a 100-fold molar excess of unlabeled IRE-fer mRNA (lanes 10 and 11), and tRNA (lanes 12 and 13), as heterologous and non-related (NR) competitors, respectively. Mock-control-labeled transcripts (IRE-fer and IRE-tvcp12) without proteins (lanes 1 and 2) were used. Other controls (lanes 3-5) were also included to show the specific interaction between labeled IRE-fer and IRE-tvcp12 with HeLa cytoplasmic extracts (lanes 3 and 4) or IRE-fer and IR trichomonad cytoplasmic extracts (lane 5). Molecular weight markers (M) in kilodaltons (kDa); arrowheads, indicate the size of protein bands cross-linked to radiolabeled RNA probes on denaturing gels detected after autoradiography; asterisk shows radiolabeled free probe. Experiments were performed three independent times with similar results. (**B**,**C**), (+) or (-) symbols indicate the presence or absence of each component on the REMSA or UV cross-linking reaction.

Then, to investigate the effect of iron on RPCs formation with the IRE-tvcp12 transcript and atypical RNA-binding proteins present in trichomonads cytoplasmic extracts under different iron conditions (Figure 5A, lanes 1 and 2), we performed UV cross-linking assays (Figure 5A, lanes 3 to 6). In IR conditions, at least one prominent ~110 kDa RNA-binding protein and two less evident protein bands of ~45 and ~30 kDa were detected cross-linked to label IRE-tvcp12 RNA (Figure 5B, lane 5). These bands showed similar sizes to some of the five (~110, ~70, ~60, ~45, and ~30 kDa) cross-linked proteins between the IRE-fer and trichomonad cytoplasmic extracts (Figure 5A, lane 3). Interestingly, in HI conditions, the IRE-tvcp12 radioactive transcript only cross-linked to very low molecular weight protein bands (<20 kDa) (Figure 5, lane 6), probably atypical RNA-binding protein degradation products, whereas with the IRE-fer transcript, no RPCs were observed (Figure 5, lane 4). This degradation should be particular for these types of proteins since the CBB-stained protein patterns of cytoplasmic extracts from both iron conditions look similar (Figure 5A, lanes 1 and 2). These results suggest that trichomonad atypical RNA-binding proteins could be degraded under HI conditions (Figure 5A, lane 6, asterisk), as occurs with the human IRP-2 [55,56].

### 3.8. The Non-Canonical IRE-tvcp12 Hairpin Specifically Interacts with Atypical Cytoplasmic RNA-Binding Proteins from Trichomonad Cytoplasmic Extracts

Moreover, to determine the specificity of the trichomonad RPCs formed between the IRE-tvcp12 RNA and trichomonad IR cytoplasmic proteins, we performed REMSA and UV cross-linking competition assays. Figure 5B shows that a single RPC was formed after this interaction (Figure 5B, lane 2), which was partially (Figure 5B, lanes 3 and 4–IRE-fer and lanes 7 and 8 –IRE-tvcp12) or completely competed (Figure 5B, lanes 5 and 6 –IRE-tvcp4) by the unlabeled probes, or not competed by the non-related RNA probe (NR; Figure 5B, lanes 9-10). As expected, no labeled band was detected in the mock experiment (Figure 5B, lane 1).

In the UV cross-linking assays (Figure 5C), the control of RNA-protein interaction without competitors showed that the IRE-tvcp12 RNA interacted with several protein bands of ~135, ~110, ~70, ~45, and ~30 kDa (Figure 5C, lane 6). Unlabeled IRE-tvcp12 RNA at 10-, 20-, and 100-fold molar excess partially (up to 85 and 90%) and completely competed with homologous labeled probes, respectively (Figure 5C, lanes 7 to 9), as compared to the control interaction without competitor (Figure 5C, lane 6). In contrast, unlabeled IRE-fer RNA at 20- and 100-fold molar excess partially cross-competed (up to 60 and 95%, respectively) with the labeled IRE-tvcp12 RNA for specific interaction with trichomonad cytoplasmic proteins (Figure 5C, lanes 10 and 11, respectively). While an excess of tRNA as NR competitor at the same molar concentrations (20- and 100-fold) had no effect (Figure 5C, lanes 12 and 13). Other controls were also included (lanes 1–5) as described in the figure legend. These results confirm the specificity of the RPCs formed between *T. vaginalis* cytoplasmic proteins and IRE-tvcp12 under IR conditions. It also shows the functionality of the IRE-tvcp12 RNA, which is a better competitor than the IRE-fer transcript, for atypical *T. vaginalis* cytoplasmic RNA-binding proteins under these experimental conditions. 

Interestingly, the protein bands crosslinked with IRE-tvcp12 have a similar size to those that interacted with trichomonad IRE-tvcp4, which were previously identified by mass spectrometry [39,40] as α-actinin 3 (~135 kDa), α-actinin 2 (~110 kDa), HSP70 (~70 kDa), and actin (~45 kDa), respectively, as part of a multiprotein complex [40]. Two of them have already been characterized as atypical *T. vaginalis* RNA-binding proteins, such as the α-actinin3 (TvACTN3) and the heat-shock protein 70 (TvHSP70) that specifically interacted with the first non-canonical IRE described in trichomonads, the IRE-tvcp4 RNA structure [39,40]. Thus, our final quest was to test whether at least these two atypical RNA-binding proteins are also part of the RNA-protein complex formed between the trichomonad cytoplasmic extracts and the IRE-tvcp12 RNA under IR conditions.

### 3.9. The Polyclonal Anti-TvACTN3r and Anti-TvHSP70r Antibodies Supershifted RNA-Protein Complex I

**Figure 6:**
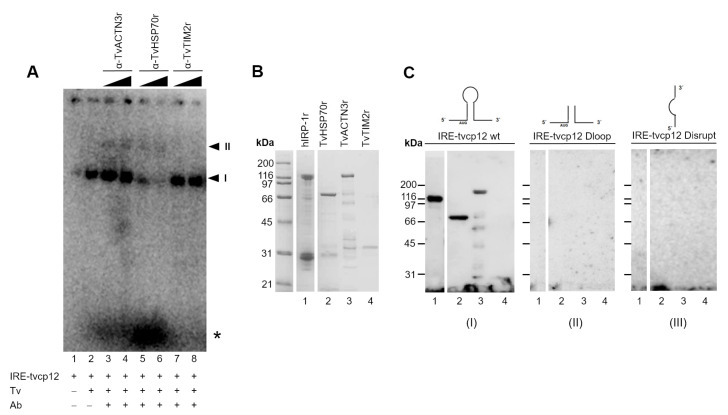
Supershift and Northwestern blot (NWB) assays show the presence and interaction of TvACTN3 and TvHSP70 in the RPC formed between *T. vaginalis* cytoplasmic extracts and IRE-tvcp12 transcript. (**A**) Supershift assay between trichomonad cytoplasmic extracts and IRE-tvcp12 in the absence (lane 2) or presence of different amounts of antibodies: lanes 3 and 4, anti-TvACTN3r; lanes 5 and 6, anti-TvHSP70r, or lanes 7 and 8, anti-TvTIM2r used as a non-related antibody. Lane 1, mock experiment. Arrowheads show the position of the RPCs I and II. Asterisk shows free radiolabeled probes. (**A**), (+) or (-) symbols indicate the presence or absence of each component on the REMSA or UV cross-linking reaction. (**B**,**C**) NWB assays using the purified recombinant proteins hIRP-1r (lane 1) used as a positive control, TvHSP70r (lane 2) and TvACTN3r (lane 3) to be tested and TvTIM2r (lane 4) as a non-related control protein. Proteins were separated by SDS-PAGE in a 10% polyacrylamide gel which was CBB-stained (**B**) or transferred onto NC membrane (**C**) and then incubated with different radiolabeled probes: IRE-tvcp12 (panel I), IRE-tvcp12 Dloop mutant (panel II), or IRE-tvcp12 Disrupt mutant (panel III).

To test our hypothesis regarding the participation of TvACTN3 and TvHSP70 in RNA-protein interactions between IRE-tvcp12 and IR trichomonad cytoplasmic extracts, we performed supershift assays using the anti-TvACTN3r and anti-TvHSP70r antibodies in REMSA reactions between parasite IR cytoplasmic extracts and the radiolabeled IRE-tvcp12 RNA transcript. Figure 6A shows that the RPC (I) (lane 2) was supershifted by the anti-TvACTN3r and the anti-TvHSP70r antisera in a concentration-dependent manner. A second RPC (II) was also observed (Figure 6A, lanes 3, 4 and lanes 5 and 6, respectively), and a reduction in the RPC (I) was also observed with the anti-HSP70 antibody. The anti-TvTIM2r antibody (at the same concentrations) used as a non-related negative control did not affect RPC formation, as expected (Figure 6A, lanes 7, 8). These results showed the specific interaction of IRE-tvcp12 with TvACTN3 and TvHSP70 present in the RPC formed with the IR trichomonad extracts.

### 3.10. The IRE-tvcp12 Binds to TvACTN3 and TvHSP70, Two Atypical RNA-Binding Proteins in Trichomonads 

**Figure 7:**
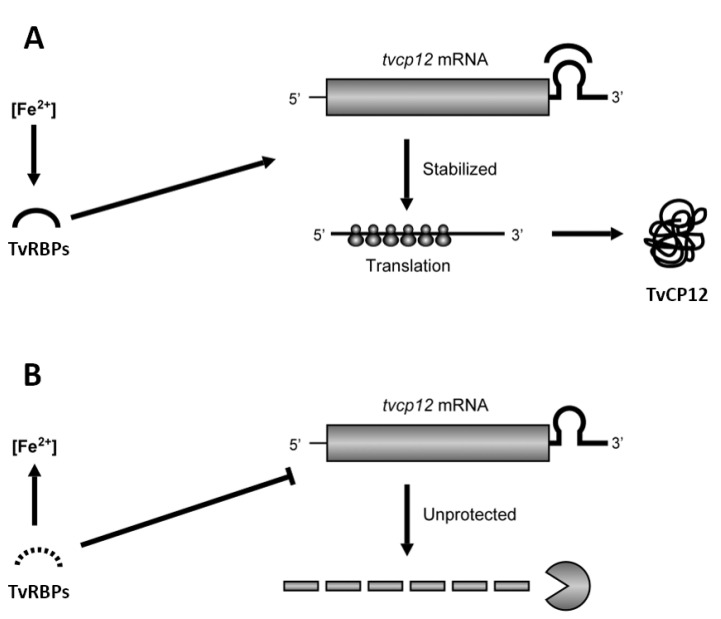
*tvcp12* gene expression as a model to show that iron-restriction upregulates gene expression at the posttranscriptional in *T. vaginalis* using a parallel mechanism mediated by RNA-protein interactions similar to the IRE/IRP system in mammals. (**A**) In iron-restricted, conditions trichomonad atypical RNA-binding proteins (TvRBP, semi-circle) bind to the IRE hairpin stem-loop structure of the *tvcp12* transcript, stabilizing its mRNA and allowing the *tvcp12* mRNA translation to occur. (**B**) In high iron conditions, the *tvcp12* mRNA is degraded by RNases due to the absence of atypical RNA-binding proteins (TvRBP dashed semicircle), which could be degraded under these iron concentrations. Thus, no translation of TvCP12 occurs.

To confirm the binding of IRE-*tvcp12* to TvACTN3 and TvHSP70, we conducted an NWB assay as another functional assay using purified TvACTN3r and TvHSP70r proteins to be tested and hIRP-1r and TvTIM2r as positive and negative control proteins, respectively (Figure 6B). Figure 6C shows that the radioactive IRE-tvcp12 transcript in the NWB assays specifically bound to TvACTN3r and TvHSP70r, as well as hIRP-1r, which was used as a positive control protein (Figure 6C, lanes 1–3). The probe did not bind to TvTIM2r used as a negative control protein (Figure 6C, lane 4, panel I). An absence of RNA-protein interactions was observed when two radiolabeled *tvcp12* deletion mutants that disrupt the loop or hairpin of the IRE-*tvcp12* RNA were used as negative control transcripts (Figure 6C, panels II and III, respectively), confirming the specificity of the interactions between IRE-tvcp12 and TvACTN3 and TvHSP70 as two of the atypical RNA-binding proteins of *T. vaginalis* that could be responsible for the trichomonad posttranscriptional regulation of the *tvcp12* mRNA by forming RPCs under IR conditions. Thus, we propose that this atypical iron regulatory mechanism at the posttranscriptional level mediated by RNA-protein interactions could control mRNA stability and the amount of translated TvCP12, depending on the iron concentrations, as described in the theoretical model (Figure 7).

## 4. Discussion

This report shows that the amount of transcript and protein TvCP12 is upregulated by the lack of iron at the posttranscriptional level due to the presence of an IRE-like hairpin stem-loop structure (IRE-tvcp12) in the 3′-UTR mRNA that interacts with atypical cytoplasmic RNA-binding proteins present in the cytoplasmic extract from trichomonads grown under IR conditions, such as TvACTN3 and TvHSP70. Thus, it is likely that expression of TvCP12 is modulated by a posttranscriptional atypical iron regulatory mechanism mediated by specific RNA-protein interactions parallel to the IRE/IRP system, preventing mRNA degradation in IR conditions as in other organisms [3,4,5,6]. 

We previously reported that the trichomonad *tvcp4* gene is upregulated by high iron at the posttranscriptional level due to the presence of a non-canonical iron-responsive element (IRE-tvcp4) at the 5′-region of the *tvcp4* transcript, blocking its mRNA translation [29]. These data suggest that iron regulates the expression of some trichomonad genes through an IRE/IRP-like system, which is parallel to the one described for iron homeostasis in mammals [3,4,5,6]. Consistent with this idea, herein we report the presence of another trichomonad non-canonical iron-responsive element, IRE-tvcp12 at the 3′-UTR of the *tvcp12* transcript (Figure 3), modulating its mRNA stability (Figure 1) through RPCs formed under IR conditions (Figure 5 and Figure 6). The *tvcp12* gene expression is upregulated by the lack of iron at the posttranscriptional level through the interaction of its hairpin-loop structure (IRE-tvcp12) with atypical cytoplasmic RNA-binding proteins under IR conditions (Figure 5 and Figure 6) that increased its mRNA half-life (Figure 1), as in mammalian transferrin receptor-1 (TfR1) mRNAs [57,58]. 

Although the *tvcp12* gene expression regulation by iron is similar to the iron regulation of the TfR mediated by an IRE/IRP system [3,4,5,6], the TfR transcript has multiple IRE structures at its 3′-UTR, and the *tvcp12* mRNA has only one non-canonical-IRE-like-structure (Figure 3). This finding is consistent with the fact that most of the trichomonad genes have short 3′-UTRs [51,59] and with other iron downregulated mRNAs with a single IRE, i.e., divalent metal transporter 1 (DMT1), CTLA4, and human cell division cycle protein 14A (CDC14A) [58,60]. 

Even if most mammalian IREs found in mRNA coding proteins involved in iron homeostasis are similar and follow a canonical stem-loop structure [61,62], the stem nucleotide sequence can vary considerably and some mutations in the loop sequence do not interfere with the binding capacity of the IRP [45]. Interestingly, the loop sequence of the non-canonical IRE-tvcp12 structure found in the *tvcp12* mRNA does not have the consensus sequence; instead, this non-canonical loop sequence (5′-UAAUUG-3′) is similar to some of the IRE-fer mutants reported by Henderson et al. [45,46]. Despite that, human IRPs bind to this non-canonical IRE-tvcp12 (Figure 4), and its loop sequence is required for RNA-binding activity to typical IRP and other HeLa proteins (Figure 4). 

Albeit the non-canonical IRE structures found in these iron differentially regulated trichomonad genes: *tvcp4* [29] and *tvcp12* are not identical, the functional assays for the IRE-tvcp12 hairpin show that the RPCs formed with HeLa and trichomonad cytoplasmic extracts are specific (Figure 4, Figure 5 and Figure 6), as those reported for the conserved canonical IRE-fer structure [45,46], or other IREs reported in nature [58,62], including the non-canonical IRE that regulates expression of alpha-hemoglobin-stabilizing protein by iron [63] and the recently described putative IREs for other iron-regulated genes in protozoan parasites such as *Entamoeba histolytica* [64] and *Giardia lamblia* [65]. 

Additionally, some of the cross-linked proteins (human IRPs or trichomonad atypical RNA-binding proteins) to IRE-tvcp12 hairpin have similar sizes to those bound to IRE-tvcp4 [29] and can be partially or completely competed with different IREs, including IRE-tvcp4. The IRE-tvcp12 hairpin is a better competitor than the canonical IRE-fer (Figure 4 and Figure 5) for RPCs formation with human IRPs or trichomonad atypical RNA-binding proteins. Moreover, our data show that the non-canonical IRE-tvcp12 structure only binds to trichomonad proteins from IR parasites (Figure 5) since iron appears to promote specific proteolytic degradation of the trichomonad RNA-binding proteins (Figure 5) by an unknown mechanism. This is an expected behavior for this type of iron regulatory mechanism despite using atypical RNA-binding proteins. Furthermore, these data confirm the presence of atypical cytoplasmic RNA-binding proteins in *T. vaginalis* only in iron-depleted conditions. Interestingly, genes encoding the canonical IRP proteins have not been found in the database of the draft of the *T. vaginalis* genome [11], suggesting that these RNA-binding proteins might also be atypical in recognizing and binding to the canonical IRE-fer sequences, as well as to the trichomonad non-canonical IRE structures [29,39]. By REMSA supershift and NWB assays, we demonstrated that at least TvACTN3 and TvHSP70 proteins are some of the atypical cytoplasmic RNA-binding proteins that *T. vaginalis* uses to regulate gene expression by iron at the posttranscriptional level. These proteins could be considered as also moonlighting multifunctional proteins in *T. vaginalis* that, in addition to its canonical properties, functions as cytoskeletal or stress response proteins, which acquire a new function as RNA-binding proteins under IR conditions that bind at least two non-canonical IRE structures in trichomonad and canonical IRE-fer of mammals.

The existence of a posttranscriptional mechanism for negative iron regulation through a parallel mechanism to the IRE/IRP system in trichomonads [29,39,40,50] could be illustrated using the upregulated *tvcp12* gene expression under IR conditions as a model. In the IR conditions (Figure 7A), trichomonad atypical RNA-binding proteins (TvRBP) bind to the IRE-tvcp12 structure (Figure 5 and Figure 6), stabilizing its mRNA (Figure 1), allowing the *tvcp12* transcript to be translated, increasing the amount of TvCP12 that relocalizes to the parasite surface (Figure 2). In contrast, in the HI conditions (Figure 7B), trichomonad atypical TvRBP are absent (possibly degraded) (Figure 5A), preventing RNA–protein interactions from occurring, allowing mRNA degradation by RNases (Figure 1C), and reducing TvCP12 synthesis (Figure 2).

The fact that this parasite could respond to the changing iron concentrations during vaginal infection by differentially regulating the expression of multiple genes at the same time supports the existence of a parallel IRE/IRP-like regulatory mechanism in *T. vaginalis,* using atypical cytoplasmic RNA-binding proteins and non-canonical IRE-like hairpin RNA structures. It also suggests its presence in the early stages of evolution using cytoskeletal (TvACTN-3) and stress response proteins (TvHSP70) instead of the typical IRP. Thus, our data suggest that the highly conserved mechanism of iron regulation mediated by the canonical IRE/IRP system might represent a specialization throughout the evolution for the maintenance of iron homeostasis [3,4,5,6,62] that started with atypical RNA-binding proteins such as actinin and heat-shock protein 70, as in *T. vaginalis*. In *T. vaginalis,* this mechanism might regulate the expression of a wide variety of genes, including some encoding proteins involved in trichomonal metabolism and virulence, in addition to those related to iron homeostasis.

## 5. Conclusions

In conclusion, in this study, we have demonstrated that *tvcp12* expression is upregulated by the lack of iron due to a differential modulation of its mRNA stability by specific RNA-protein interactions under IR conditions mediated by the binding of a non-canonical IRE-tvcp12 hairpin stem-loop structure located at the 3′-UTR of the *tvcp12* mRNA to TvACTN3 and TvHSP70 atypical cytoplasmic proteins. 

By combining the data from *tvcp4* [29,39,40] and *tvcp12* gene expression regulation by iron, and controlling translational blockage or mRNA stability, the existence of a posttranscriptional mechanism through a parallel IRE/IRP-like system in *T. vaginalis*, an early branching protozoan, can be illustrated. This regulatory mechanism could explain the way some trichomonad genes are differentially expressed by the same iron concentration at the posttranscriptional level. This will pave the way to enhance our understanding of trichomonad gene expression regulation in response to iron variations during infection. We are currently working on identifying the presence of IRE-like structures at the 3′-UTR of mRNAs upregulated by iron-restricted conditions and their impact on their gene expression regulation at the posttranscriptional level.

## Data Availability

The data presented in this study are available upon request to the corresponding author.
